# Information Theory Filters for Wavelet Packet Coefficient Selection with Application to Corrosion Type Identification from Acoustic Emission Signals

**DOI:** 10.3390/s110605695

**Published:** 2011-05-27

**Authors:** Gert Van Dijck, Marc M. Van Hulle

**Affiliations:** Computational Neuroscience Research Group, Katholieke Universiteit Leuven, Herestraat 49, B-3000 Leuven, Belgium; E-Mail: marc.vanhulle@med.kuleuven.be

**Keywords:** acoustic emission, chemical process industry, corrosion monitoring, feature subset selection, information theory, mutual information, Wavelet Packet Transform

## Abstract

The damage caused by corrosion in chemical process installations can lead to unexpected plant shutdowns and the leakage of potentially toxic chemicals into the environment. When subjected to corrosion, structural changes in the material occur, leading to energy releases as acoustic waves. This acoustic activity can in turn be used for corrosion monitoring, and even for predicting the type of corrosion. Here we apply wavelet packet decomposition to extract features from acoustic emission signals. We then use the extracted wavelet packet coefficients for distinguishing between the most important types of corrosion processes in the chemical process industry: uniform corrosion, pitting and stress corrosion cracking. The local discriminant basis selection algorithm can be considered as a standard for the selection of the most discriminative wavelet coefficients. However, it does not take the statistical dependencies between wavelet coefficients into account. We show that, when these dependencies are ignored, a lower accuracy is obtained in predicting the corrosion type. We compare several mutual information filters to take these dependencies into account in order to arrive at a more accurate prediction.

## 1. Introduction

### 1.1. Corrosion Monitoring

A large part—25 to 40%—of the costs related to corrosion can be saved by the use of appropriate corrosion monitoring and control systems [1]. Corrosion monitoring provides feedback to operators about the state of the plant, information that in principle can be used for reducing the costs due to corrosion [1]. Direct costs can be avoided thanks to the increased reliability of the plant, avoidance of the disruption of the supply of products, decreased loss of capital and avoidance of lawsuits against companies (e.g., due to pollution caused by leaks of the installations), among other factors. Indirect costs can be equally important as these costs have an impact on the society and the environment. In some sectors, damage due to corrosion can be tolerated, but in the chemical, petrochemical and nuclear sectors, corrosion damage can be catastrophic, even resulting in the loss of lives and irreversible environmental damage.

Regular practice in the chemical process industry consists of periodic inspections of the plant, e.g., every 3 months, every 6 months or every year [2]. A recurring problem with such periodic inspections is that one can overlook the active damage that occurs in the plant; furthermore, immediately after inspection, the damage can continue to grow until the next periodic inspection is scheduled. Clearly, such cases should be avoided. A solution is offered by continuous monitoring procedures using corrosion monitoring systems. Different techniques are available for corrosion detection and monitoring in the chemical process industry [2,3]. In this research, we detect the most important types of corrosion in the chemical process industry from acoustic emission signals that are emitted during the corrosion process. Chemical reactions, as occurring during corrosion, emit acoustic activity [4,5] as well as the microscopic damage and fracture processes resulting from corrosion [6]. The acoustic emission technique has the advantage that it is low cost and allows for a continuous, on-line monitoring so that the damage can be detected as soon as it occurs [3].

### 1.2. Importance to Distinguish between Different Corrosion Types

The most frequent corrosion processes in the chemical process industry are: uniform corrosion (or general corrosion), pitting and stress corrosion cracking (SCC) [1,2]. It may also be possible that no corrosion process is active during the measurement. Therefore, we consider, in addition to the mentioned types, the absence of corrosion also needs to be discriminated.

There are at least two important reasons why researchers and industrial experts should be able to distinguish between different types of corrosion. Firstly, pitting and SCC are more harmful types of corrosion compared to uniform corrosion. Uniform corrosion reduces the thickness of the material relatively uniformly, hence taking a long time before holes are formed in the material. On the other hand, pitting causes pits and SCC causes cracks which can grow much faster, puncturing the material. This may lead to unexpected leaks in chemical plants. Therefore, occurrence of pitting and SCC Acoustic Emission (AE) events should advance the inspection of the installation.

Secondly, the discrimination between different corrosion processes should be performed prior to the quantitative analysis of correlating acoustic emission activity to the corrosion rate. In Seah *et al.* [7] a quantitative analysis has shown that the count rate (defined by the authors as the total number of threshold crossings of AE signals per unit area of the exposed part of the metal sample and per unit time) is correlated with the rate of corrosion measured by means of the weight loss of the metal sample. A quantitative relation between the number of AE events and the number of pits in pitting as well with the pitted area and volume was established in Mazille *et al.* [8]. In stress corrosion cracking, a relationship between AE parameters (counts change per unit time and energy change per unit time) and the corrosion speed (change of crack length per unit time) has been established [9]. This shows that for several corrosion processes one can estimate the corrosion speed from AE parameters, although one should first link an AE event to the corresponding corrosion process. Erroneously relating AE events originating from pitting to SCC leads to a poor estimate of the corrosion speed of SCC and *vice versa*.

### 1.3. Wavelet Packet Feature Extraction and Selection from Acoustic Emission

Although future successes in corrosion prevention will still depend on selecting and developing more corrosion resistant materials, it is expected that the main progress in corrosion prevention will be achieved with better information-processing strategies and the development of more efficient monitoring tools that support corrosion control programs [10]. Feature extraction, feature subset selection, and classifier choice and design are all information-processing strategies that should be explored in the design of better corrosion monitoring systems.

Features to characterize the acoustic emission activity have often been obtained in the time-amplitude domain [2,5,11], the frequency domain [2,5,12], or the time-frequency domain using the Continuous Wavelet Transform (CWT) [13,14], the Discrete Wavelet Transform (DWT) [14] or the Wavelet Packet Transform (WPT) [15]. The process of constructing informative features that can help to discriminate between different classes is not trivial, but some generic approaches are available [16]. One of those approaches is to consider basis functions that can be used to extract features. A library of basis functions can be obtained from the Wavelet Packet Transform [17–19].

A challenge that arises after the extraction of wavelet coefficients with a Wavelet Packet Transform is the selection of a basis that is optimal in some sense, or the selection of a few coefficients for signal compression or pattern recognition purposes [18,20–22]. One of the most established algorithms to select wavelet coefficients for the prediction of a target variable, the corrosion type in this case, is the local discriminant basis (LDB) algorithm [20–22]. In previous research, we pointed to some disadvantages of the LDB algorithm [15]; in particular, the statistical dependency between wavelet coefficients, since it is leading to a lower prediction accuracy, but we did not come up at the time with a remedy to overcome it.

In the research reported in this article, we contribute to the selection of the most informative basis functions, from a library of wavelet packets, to distinguish between different types of corrosion, using information-theoretic criteria. We use the mutual information [23] to guide the search for informative basis functions by taking into account the statistical dependencies between the wavelet coefficients. The advantage of using the mutual information is that it easily enables us to take dependencies between features into account, *i.e.*, the wavelet coefficients in our case [24]. Moreover, the behavior of mutual information in feature selection is well-understood [24].

## 2. Materials and Methods

### 2.1. Signal Acquisition

**Figure 1.**
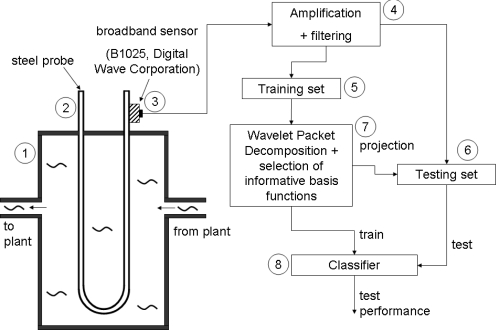
Processing stages for making predictions of the corrosion type. A steel probe (2) is inserted in a bypass (1) of the chemical process plant and is therefore exposed to the same environmental conditions as the installation. Acoustic events are captured by means of a broadband sensor (3). Subsequently AE signals are amplified and filtered (4). In order to obtain a fair validation of the system, the acquired signals are split into a training (5) and testing set (6). Features are extracted from the training signals by means of a Wavelet Packet Decomposition (7). A classifier (8) is trained based on the selected wavelet coefficients of the training set. Testing signals are projected onto the selected basis functions. Subsequently, the wavelet coefficients of the testing signals are used to test the overall performance of the system.

This section describes the experimental set-up to obtain the acoustic emission signals. A U-shaped steel sample is shown in Figure 1.

The probe is designed such that the corrosion process occurring in the probe is representative for that in the plant [2]. Therefore, the probe is made of the same type of steel as the plant and the probe is exposed to the same environmental conditions, that is the same corrosive medium, temperature and pressure. This is represented in Figure 1 by means of the input flow that arrives from the plant and the output flow that is guided back to the plant, hence, forming a bypass of the process plant. Measuring the corrosion with a reference probe is based on some important considerations. The probe is relatively small: approximately 30 cm in height. This means that dampening of the waves when they propagate over such small distances is small. On the other hand when performing measurements on the large installation itself, AE waves may have dampened out before they reach a sensor when there is no sensor in the neighborhood of the AE source. This would call for a dense network of AE sensors, leading to a more complex and expensive set-up. Moreover, due to the large difference in distances that waves could have travelled, AE events can be deformed to different degrees e.g., due to dispersion. This deformation will hamper the recognition of the type of corrosion from the waveforms. Thirdly, installations are often exposed to external sources that can create AE events: e.g., mechanical vibrations, rain drops, *etc.* These sources may be confounded with AE events originating from corrosion events.

The damage that occurs on the probe is captured by means of piezoelectric sensors attached to the corroding probe. In order to guarantee a good acoustical transfer from the probe to the sensor, a ‘high vacuum’ grease (DOW Corning^®^) is applied between the sensor and the probe. The sensors used here are broadband sensors (B1025, Digital Wave Corporation) [2]. These sensors have a guaranteed frequency bandwidth from 50 kHz to 2 MHz and can be used in a temperature range from −50 °C to 100 °C. Subsequently, the signals are amplified with an amplification factor of approximately 40 dB. The signals are then bandpass filtered between 50 kHz–2 MHz, because outside this range the sensor does not guarantee reliable information. Signals are sampled at 20 MHz or 25 MHz, both sampling rates are safely higher than the Nyquist sampling rate of 4 MHz for signals up to 2 MHz. Before computing the wavelet transform, signals are resampled to the same sampling rate (25 MHz) if they were sampled at 20 MHz.

### 2.2. Experimental Conditions

Two types of steel that belong to the most often used construction materials in the chemical process industry [1] are considered: carbon steel and stainless steel. The carbon steel considered here is German Material Number 1.0038, called S235JRG2 (DIN EN 10025) or RSt 37-2 (DIN 17100). The stainless steel considered here is German Material Number 1.4541, called X6CrNiTi18-10 (DIN EN 10088-2) and similar to AISI 321. The chemical composition of the two considered steel types can be found in [25]. All materials and experimental conditions are summarized in Table 1, together with the number of different experiments for the material-environment combinations (the environment is the combination of a corrosive medium and a temperature). The total number of time series obtained from these experiments is indicated between parentheses. The signals for each experiment were often collected over several days to obtain a representative set of signals. The acoustic emission data set contains 197 time series of “absence of corrosion” (indicating that no corrosion was active during these experiments), 194 time series of uniform corrosion, 214 time series of pitting and 205 time series of SCC. The time series have been assigned a corrosion class label by an expert [2] based on a visual inspection of the damage to the probe, the experimental conditions, and the inspection of the acoustic emission signals [2]. Each time series consists of “N” = 1,024 samples.

**Figure 2.**
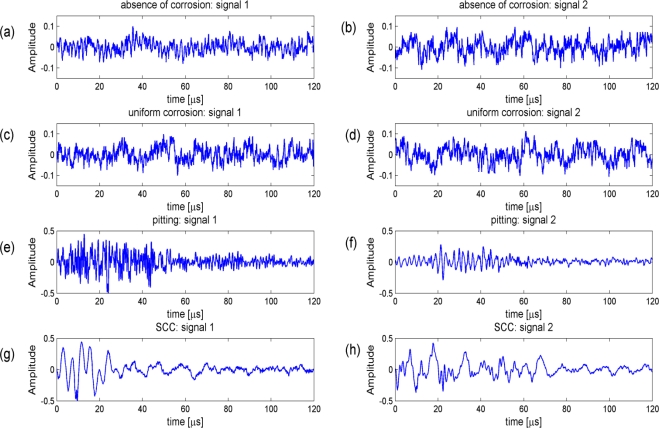
Example signals of different corrosion types. The example of the absence of corrosion in **(a)** was captured from stainless steel in CaCl_2_ 40 weight% at 85 °C environment. The example of the absence of corrosion in **(b)** was captured from carbon steel NaOH 20 weight% + NaCl 3 weight% at 80 °C environment. The examples in **(c)** and **(d)** are from continuous emissions during uniform corrosion of carbon steel in H_3_PO_4_ 10 weight% at environment temperature. The signals in **(e)** and **(f)** are burst emission pitting signals captured from stainless steel in brackish water + FeCl_3_ 1 weight% at 45 °C environment. In **(g)** a SCC burst emission signal was captured from stainless steel in CaCl_2_ 40 weight% at 85 °C environment; **(h)** SCC burst emission signal was captured from carbon steel Ca(NO_3_)_2_ 60 weight% at 105 °C environment.

The different mechanisms that lead to the emission of acoustic events have been treated extensively in [2,6,15]. In Figure 2, we show some examples of different acoustic signals that were captured during different corrosion processes. Acoustic signals in the uniform corrosion experiments are characterized by a continuous-type acoustic emission signal [2,15], see also Figure 2(c,d). Localized forms of corrosion, such as pitting and stress corrosion cracking, lead to a burst-type acoustic activity, see Figure 2(e,f) and Figure 2(g,h) respectively.

## 3. Wavelet Packet Decomposition

The basic approach for constructing features is to compute a number of general statistical parameters from time series such as the median, the mean, the standard deviation and higher-order moments. However, when restricting oneself to a limited number of parameters in advance, important information may be lost due to the implicit assumptions behind these parameters, e.g., the mean and standard deviation are only sufficient to characterize signals that consist of independent and identically distributed (i.i.d.) Gaussian noise.

A more thorough approach is to extract the wavelet coefficients from a wavelet packet decomposition (WPD) [26,27]. Wavelet packet decompositions offer a library of templates that have many desirable properties. First of all, WPD’s are founded on a solid mathematical theory [27] that allows one to represent the signals in a new basis. The decomposition in a new wavelet packet basis guarantees that no ‘information’ is lost as the original signals can always be reconstructed from the new basis. Secondly, the templates in a wavelet packet decomposition can be interpreted in terms of frequencies and bandwidths [27]. Thirdly, wavelet packet decompositions are more flexible than the discrete wavelet transform and the Fourier transform. This means that the basis functions that are used in a discrete wavelet transform (DWT) are also available in the wavelet packet decomposition. We will use the wavelet coefficients, obtained from a wavelet packet decomposition, as the constructed features.

### 3.1. Wavelet Packet Decomposition Basics

The reader acquainted with wavelet packet decompositions may skip this section, which introduces the background to feature extraction from wavelet packet decompositions. This background is needed in order to understand the feature selection procedures in Sections 3.2 and 4. We will use the terminology of template and basis function interchangeably. Strictly speaking, a template is a more general terminology, because it does not need to be part of a basis.

We represent a single time series by means of a sequence of observations x(t): x(0), x(1), ... x(N-1), where ‘t’ refers to the time index and ‘N’ is the number of samples. The time series x(t) can be considered as being sampled from an ‘N’ dimensional distribution defined over an N dimensional variable X(t): X(0), X(1), ... X(N-1), we write this ‘N’ dimensional variable in short hand notation as **X**_0:N−1_.

Features are computed from a wavelet packet decomposition by computing the inner product between the templates and the time series (using a continuous representation, for the ease of notation):

(1) $$\gamma_{i,j,k}=\langle x(t),\Psi_{i}^{j}(t-2^{i}k)\rangle =\int_{-\infty }^{+\infty }x(t)\Psi_{i}^{j}(t-2^{i}k)\;dt$$

A feature, in this case a wavelet coefficient, in the wavelet packet decomposition needs to be specified by the scale index ‘i’, frequency index ‘j’ and time index ‘k’. The coefficient γ_i,j,k_ can be considered as quantifying the similarity, by means of the inner product, between time series x(t) and wavelet function *Ψ_i_^j^* (*t* – 2*^i^k*) at position 2*^i^k* in time. The parameter ‘i’ is the scale index and causes a dilation (commonly called a ‘stretching’) of the wavelet function *Ψ^j^* (*t*) by a factor 2^i^:

(2) $$\Psi_{i}^{j}(t)=\frac{1}{\sqrt{2^{i}}}\Psi^{j}(\frac{t}{2^{i}})$$

**Figure 3.**
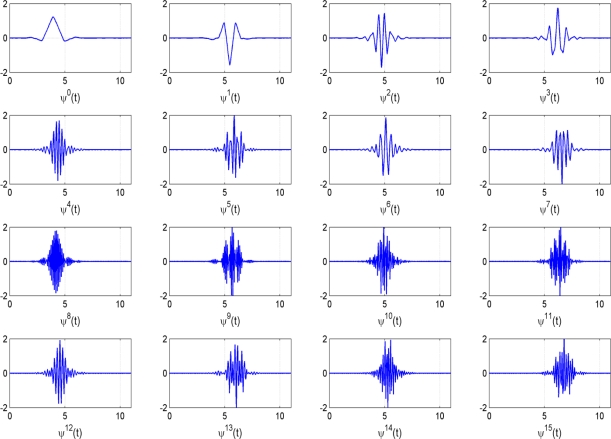
Templates (wavelet packets) corresponding to the 12-tap Coiflet filter.

It is the parameter ‘j’ that determines the shape of the template. If we choose the 12-tap Coiflet filter [20], we obtain the first 16 different templates *Ψ*^0^(*t*), *Ψ*^1^(*t*), *Ψ*^2^(*t*),... *Ψ*^15^(*t*) shown in Figure 3. This 12-tap Coiflet filter has been consistently used in the experiments in Section 5. The construction of these basis functions can be found in text books [27]. The shapes of these basis functions also motivate the use of wavelet packet decompositions in our application. With an appropriate scaling and time shift some of the basis functions in Figure 3 resemble the AE bursts in Figure 2 (e–h). Choosing the appropriate template, the scaling factor and the time shift is the task of the feature selection procedure in Section 4.

**Figure 4.**
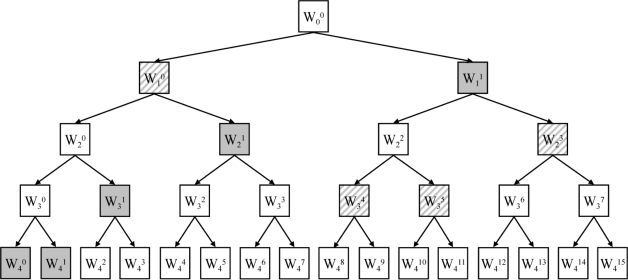
Library of wavelet packet functions. Different subspaces are represented by W_i_^j^. Index ‘i’ is the scale index, index ‘j’ is the frequency index. The depth ‘I’ of this tree is equal to 4. Every subtree within this tree, where each node has either 0 or 2 children, is called an admissible tree. Two admissible trees are emphasized, one shaded in grey and one marked with diagonals.

In Figure 4, we show a graphical representation of the different subspaces that are obtained in a wavelet packet decomposition. In the discrete wavelet transform, the only nodes in the tree that are considered are W_1_^1^, W_2_^1^, W_3_^1^, W_4_^1^ and W_4_^0^ these subspaces are shaded in grey.

The first four subspaces are spanned by ${\left\{\psi_{1}^{1}(t-2k)\right\}}_{k\in 𝕑}$, ${\left\{\psi_{2}^{1}(t-2^{2}k)\right\}}_{k\in 𝕑}$, ${\left\{\psi_{3}^{1}(t-2^{3}k)\right\}}_{k\in 𝕑}$, and ${\left\{\psi_{4}^{1}(t-2^{4}k)\right\}}_{k\in 𝕑}$ respectively. Subspace *W_4_^0^* is spanned by ${\left\{\psi_{4}^{0}(t-2^{4}k)\right\}}_{k\in 𝕑}$. So in the discrete wavelet transform, the signals are only analyzed by means of the time translated functions of $\Psi_{4}^{0}(t)$. Note that $\Psi_{0}^{0}(t)$ is called the scaling function, shown as the first template in Figure 3, and the dilated and time translated functions of $\Psi_{0}^{1}(t)$ (the latter is called the mother wavelet function and is shown as the second template in the top row of Figure 3). In Figure 4, only two bases are shown: the gray shaded basis corresponds with the discrete wavelet transform, the basis marked with diagonals is chosen arbitrarily and is one of the possible bases in the wavelet packet decomposition. The basis marked with diagonals puts more emphasis on a finer analysis of the higher frequency part of the signals.

Retaining any binary tree in Figure 4, where each node has either 0 or 2 children, leads to an orthonormal basis for finite energy functions, denoted as *x(t)* ∈ *L^2^(*ℝ*)*:

(3) $$\int_{-\infty }^{+\infty }|x(t){|}^{2}dt<\infty$$

Such a tree is called an admissible tree. If the leaves of this tree are denoted by {*i_l_, j_l_*}_1 ≤_ *_l_* _≤_ *_L_* the orthonormal system can be written as:

(4) $$W_{0}^{0}={⊕}_{l=1}^{L}\;W_{il}^{jl}$$

This means that the space *W_0_^0^*, which is able to represent the input space of the time series, can be decomposed into orthonormal subspaces *W_il_^jl^*.

It should be noted that a full wavelet packet decomposition yields too many features. In cases where one can assume that the exact time location ‘k’ of the template is of no importance, one can, e.g., consider an average or the energy of wavelet coefficients over time for each possible combination of the scale index ‘i’ and the frequency index ‘j’. This will lead to fewer features to be selected from. Here, we will consider the full complexity of the problem, when the exact time location of the template can be of importance, and consider all coefficients from a full wavelet packet decomposition as selectable.

A full wavelet packet decomposition leads to N × (log_2_N + 1) features. This can be seen as follows. From Figure 4, it can be noted that the number of subspaces at a certain scale ‘i’ is determined by the scale index ‘i’. The number of subspaces at scale ‘i’ is equal to 2^i^. Therefore the frequency index ‘j’ at a certain scale ‘i’ will be an integer from [0, 2^i^ − 1], indicating the starting position of the subspace at scale ‘i’.

As can be seen from Equation (1), at scale ‘i’, the inner products are computed at discrete time instants *2^i^k*. Therefore, at scale 0, we obtain ‘N’ (length of the signals) coefficients: γ_0,0,0_, ... γ_0,0,N−1_. At the next scale, ‘i’ = 1, we obtain N/2 coefficients in each subspace *i.e.*, γ_1,0,0_, ... γ_1,0,N/2−1_ and γ_1,1,0_, ... γ_1,1,N/2−1_.

At the highest frequency resolution ‘i’ = log_2_N, and we obtain coefficients: γ_log_ *_N_*_,0,0_, ... γ_log_ *_N_*_,_*_N_*_−1,0_. Hence, at each scale, there are ‘N’ coefficients, and in total there are log_2_N+1 different scale levels. This leads overall to N × (log_2_N + 1) different coefficients to select from. The variables that can be associated with the coefficients *γ_i,j,k_* are further denoted by capitals *Γ_i,j,k_*.

### 3.2. Local Discriminant Basis

In this section, we consider the selection of the most discriminative basis functions *Ψ_i_^j^* (*t* – 2*^i^k*) in order to make a prediction about the target variable ‘*y*’ (*i.e.*, the corrosion class). The target variable is a class variable taking values 1 ... *#C*, where *#C* is the total number of classes. An outline of the Local Discriminant Basis algorithm [22] is provided. We assume that we are given a set of training signals *x_j_* and, for each one of them, we are given the associated target class *c_j_*:{(*x_j_*,*c_j_*)}.

Step 0: Expand each training signal into a time-frequency dictionary D: this involves the computation of all coefficients *γ_i,j,k_* for each training signal, and assumes that we choose a particular conjugate mirror filter [27] in advance, which will define the templates.
Step 1: Estimate the class conditional probability density functions *p̂^y^* (*Γ_i,j,k_*) (PDF’s) for each wavelet coefficient variable, *Γ_i,j,k_*, in the dictionary. Superscript ‘*y*’ refers to the class label, with *y* = 1, 2, ... *#C* and *#C* is the total number of classes. These PDF’s were estimated by means of the averaged shifted histograms method (ASH) as in Saito *et al.* [22].
Step 2: For each wavelet coefficient variable, *Γ_i,j,k_*, compute the discriminant measure *δ_i,j,k_*. The computational cost of this procedure is O((N+1)log_2_N). Many discriminant measures can be used in practice. We use the symmetric relative entropy, Equation (5), as in Saito *et al.* [22]. The relative entropy for *Γ_i,j,k_* between two classes, *y* = 1 and 2, can be computed as [23]:
  (5) $$D({\hat{p}}^{1}(\Gamma_{i,j,k}),{\hat{p}}^{2}(\Gamma_{i,j,k}))≜\int {\hat{p}}^{1}(\gamma_{i,j,k})\text{log}\frac{{\hat{p}}^{1}(\gamma_{i,j,k})}{{\hat{p}}^{2}(\gamma_{i,j,k})}d\gamma_{i,j,k}$$

Because this discriminant measure is not symmetric, a symmetric version is obtained as:

(6) $$\delta_{i,j,k}=D^{s}({\hat{p}}^{1}(\Gamma_{i,j,k}),{\hat{p}}^{2}(\Gamma_{i,j,k}))=D^{s}({\hat{p}}^{1}(\Gamma_{i,j,k}),{\hat{p}}^{2}(\Gamma_{i,j,k}))+D^{s}({\hat{p}}^{2}(\Gamma_{i,j,k}),{\hat{p}}^{1}(\Gamma_{i,j,k}))$$

When more than two classes are considered, *δ_i,j,k_*, is defined as the sum over all (*#C*.(*#C* − 1))/2 pairs of different classes as:

(7) $$D_{Pair}^{S}({\hat{p}}^{1}(\Gamma_{i,j,k}),{\hat{p}}^{2}(\Gamma_{i,j,k}),\ldots ,{\hat{p}}^{c}(\Gamma_{i,j,k}))=\sum_{m=1}^{\#C-1}\sum_{n=m+1}^{\#C}D^{s}({\hat{p}}^{m}(\Gamma_{i,j,k}),{\hat{p}}^{n}(\Gamma_{i,j,k}))$$

Step 3: Evaluate the discriminant power of each basis *B* ∈ *D* (the dictionary) and obtain the best basis *Ψ* for which the discriminant power is maximal:
  (8) $$\Psi =\underset{B\in D}{\text{arg}\;\text{max}}\sum_{(i,j,k)\in B}\delta_{i,j,k}$$

Hence, one searches for the indices (*i,j,k*) such that the associated basis functions form a basis B. This corresponds also with the search for an admissible tree in Figure 4, with the largest discriminant power.

Step 4: Select ‘*m*’ basis functions, *Ψ_i_^j^* (*t* – 2*^i^k*), from *Ψ* corresponding to the ‘*m*’ largest *δ_i,j,k_*. The number of basis functions ‘*m*’ to be retained is not determined in Saito *et al.* [22]. Therefore, we perform experiments for ‘*m*’ ranging from 1 to 50 basis functions.
Step 5: Construct classifiers using the ‘*m*’ coefficients, *γ_i,j,k_*. Experiments with different classifiers are performed in Section 5.

In Step 3, the algorithm searches a basis *Ψ* for which the discriminant power is maximal. However, the total discriminant power in Step 3 is computed as the sum of the discriminant measures of each of the coefficients in a basis *B*: Σ_(_*_i,j,k)_*_∈_*_B_* δ*_i,j,k_*.

The additive property of the discriminant powers of coefficients in a basis leads to a very rapid search for the basis with the highest discriminant power. It is easily seen that an optimal basis can be found in O(*N*) comparisons, with ‘*N*’ the length of the signal, see Mallat [27]. However, as we showed before [15], the sum of the discriminant measures in Equation (8) does not necessary reflect the joint discriminant power, *i.e.*, taking the joint probability distribution of the wavelet coefficients into account. It will only be the case when the wavelet coefficients are class conditional independent [15]. When some wavelet coefficients are highly correlated, they may capture essentially the same information and, hence, the joint discriminant power is not simply a sum of the marginal discriminant measures. The consequence is that the accuracy in classification prediction may increase at a much slower rate compared to the case when the dependencies between the coefficients are taken into account. This is exactly what we will show in Section 5. So far we did not present a solution to take the dependencies into account. In Section 4, we present information theoretic filter feature selection approaches to serve this purpose.

### 4. Information Theory Filter Feature Selection Approaches

The feature selection procedures based on the mutual information are called filter approaches, due to the fact that the classifier used in the prediction is not involved in the selection of the features [28]. An alternative approach is the wrapper approach [28] in which the classification algorithm is involved in the selection of the features. The wrapper approach is often computationally more expensive, but may lead to a higher classification accuracy. A follow-up paper that combines a wrapper approach and a filter approach in a so called hybrid filter-wrapper approach is in preparation. The reason to use mutual information here is that it is a well-established criterion for taking dependencies between variables or features into account [24]. The high dimensional mutual information between a feature vector **F** and class variable C can be defined as:

(9) $$MI(F;C)=\sum_{c=1}^{\#C}\int_{F}P(f,c)\log_{2}(\frac{P(f,c)}{P(f)P(c)})df$$

We perform a sequential forward search (SFS) over all wavelet coefficients using a mutual information criterion. In the SFS, we start with the empty feature set S = {Ø} as the selected coefficients so far and the whole dictionary D = {Γ_i,j,k_}, with 0 ≤ i ≤ log_2_N, 0 ≤ j ≤ 2^i^ – 1 and 0 ≤ k ≤ N/(2^i^) – 1, as the available feature set. In each iteration of the SFS, the variable Γ’_i,j,k_, which achieves the highest value of the mutual information criterion, taking into account the previously selected features, is selected. S is updated in each iteration as: S = S ∪ Γ’_i,j,k_ and the dictionary is updated as D = D\{Γ’_i,j,k_}. Three different mutual information criteria were compared for the SFS filter: a density-based method (Section 4.1), a distance-based method (Section 4.2) and a relevance-redundancy method (Section 4.3).

### 4.1. Parzen Window Density (MI Parzen)

The estimation of the mutual information by means of a Parzen window density estimator was proposed in [29]. This is a probability density based mutual information estimator. If a Gaussian window function is used, the mutual information is estimated as (a hat is used to indicate an estimator):

(10) $$\begin{array}{c} \hat{MI}(F;C)=\hat{H}(C)-\hat{H}(C|F)\;\text{with}\\ \hat{H}(C|F)=-\sum_{j=0}^{n}\frac{1}{n}\sum_{c=1}^{\#C}\hat{p}(c|f_{j}){\text{log}}_{2}\hat{p}(c|f_{j}) \end{array}$$

(11) $$\hat{p}(c|f)=\frac{\sum_{j\in I_{c}}\text{exp}{\left(-(f-f_{i}\right)}^{T}\sum^{-1}(f-f_{i})/2h^{2})}{\sum_{k=1}^{C}\sum_{j\in I_{k}}\text{exp}(-{\left(f-f_{i}\right)}^{T}\sum^{-1}(f-f_{i})/2h^{2})}$$

The functional H(.) is the entropy [23]. Further, I_k_ is the set of indices of data points which belong to class “k”, **f**_j_ is the feature vector of the j’th training data point and #C is the total number of classes. The covariance matrix **Σ** is estimated as the full sample covariance matrix. The parameter “h” is set to a default value as suggested in the experiments in [29]: h = 1/log_2_(n), where “n” is the sample size of the training set. This estimator is referred to as “MI Parzen”.

### 4.2. K-Nearest Neighbors (MI knn)

Instead of estimating the probability density functions, the mutual information between a discrete class variables and a feature vector **F** can be estimated based on the pairwise distances between data points. We presented such an approach for feature selection, in case of a discrete target variable, in [30]. The mutual information estimator relies on the Kozachenko-Leonenko entropy estimator [31] of the differential entropy:

(12) $$\hat{H}(F|c)=-\psi (k)+\psi (n_{c})+\text{ln}(c_{d})+\frac{d}{n_{c}}\sum_{i\in I_{c}}\text{ln}({ɛ}_{c}(i,k))$$

which is plugged into:

(13) $$\hat{MI}(F;C)=\hat{H}(F)-\sum_{c=1}^{\#C}\hat{H}(F|c)\hat{p}(c)$$

In Equation (12), ψ(.) is the psi-function, “n_c_” the number of training data points in class “c”, ɛ_c_(i,k) is twice the distance from the i’th data point in class “c” to its k’th neighbor in class “c” in the training set, “d” the dimensionality of the data points and “c_d_” the volume of the d-dimensional unit ball. We used the Euclidean distance between data points, in this case “c_d_” = π^d/2/^Γ(1 + d/2), with Γ(.) the gamma-function.

The unconditional entropy Ĥ(**F**) in Equation (13) can be estimated similarly as the conditional entropy in Equation (12), but with “n_c_” replaced with the total number of training points “n” and ɛ_c_(i,k) replaced by ɛ(i,k), *i.e.*, twice the distance from data point “i” to its “k” nearest neighbor when all training data points from all classes are merged into one set. The prior probabilities *p̂*(*c*) are estimated as the number of training points in class “c” divided by the total number of training points as follows: n_c_/n. In the experiments, the number “k” of nearest neighbors was set equal to 6. This estimator is referred to as “MI knn”.

### 4.3. Relevance-Redundancy Approach

Relevance-redundancy approaches select features that are highly relevant with respect to the class variable, but penalize a feature if it is redundant with respect to previously selected features. These approaches often use mutual information to estimate both the relevance and the redundancy. Suppose that F_i_ is a candidate feature to be selected and that S is the set of already selected features; a relevance-redundancy criterion based on the normalized mutual information [32] is then obtained as:

(14) $${\text{Crit}}_{S}(F_{i},C)=MI(F_{i};C)-\frac{1}{\left|S\right|}\sum_{F_{S}\in S}\frac{1}{\min\{H(F_{i}),H(F_{S})\}}MI(F_{i};F_{S})$$

where |S| is the size of the set of already selected features. Note that, as opposed to Equations (11) and (13), here only the lower dimensional MI(F_i_;C) and MI(F_i_;F_s_) are required. Note that the normalization in Equation (14) is achieved by dividing MI(F_i_;F_s_) through min{H(F_i_), H(F_s_)}. The ratio $\frac{1}{\text{min}\{\text{H}({\text{F}}_{\text{i}}),\text{H}({\text{F}}_{\text{S}})\}}MI({\text{F}}_{\text{i}};{\text{F}}_{\text{s}})$ will be a value between 0 and 1, because MI(F_i_;F_s_) is always smaller or equal to the minimum of *H*(*F_i_*) and *H*(*F_s_*), hence, this ratio is called the normalized mutual information [32]. In Equation (14), the mutual information MI(F_i_;C) quantifies the relevance of feature F_i_ with respect to the target variable ‘C’, it will be large when F_i_ is highly relevant. The term $\frac{1}{\left|S\right|}\sum_{F_{S}\in S}\frac{1}{\text{min}\{\text{H}({\text{F}}_{\text{i}}),\text{H}({\text{F}}_{\text{s}})\}}MI({\text{F}}_{\text{i}};{\text{F}}_{\text{s}})$ quantifies the redundancy of F_i_ with the already selected features F_s_∈S. When F_i_ and F_s_ are strongly dependent, or correlated in a more stricter sense, $\frac{1}{\text{min}\{\text{H}({\text{F}}_{\text{i}}),\text{H}({\text{F}}_{\text{s}})\}}MI({\text{F}}_{\text{i}};{\text{F}}_{\text{s}})$ will be large, hence the relevance term in Equation (14) MI(F_i_;C) will be penalized. This allows features that are less relevant, but have a very low redundancy with the already selected features, to be included.

In the computation of the normalized mutual information, the features were first discretized into 3 states [33]: values of F_i_ < μ(F_i_) – (σ(F_i_))/2 were set to state 0, μ(F_i_) – (σ(F_i_))/2 ≤ F_i_ ≤ μ(F_i_) + (σ(F_i_))/2 were set to state 1 and values of F_i_ > μ(F_i_) + (σ(F_i_))/2 were set to state 2. Note that μ(F_i_) and σ(F_i_) are, respectively, the mean and standard deviation of F_i_. The mutual information was then computed from the contingency tables of the discretized features, *i.e.*, from the co-occurrences of the states of different features.

## 5. Results and Discussion

We tested four different popular classifiers to predict the different corrosion types:

- k-nearest neighbor (knn): the Euclidean distance is used with “k” set to 3, see Section 4.5.4 in [34] for a reference on k-nearest neighbor classification;
- decision tree J48 (WEKA’s implementation of C4.5) from WEKA package 3.4.1 [35], we used the default values from the WEKA package, *i.e.*, the minimum number of instances per leaf (-M) equal to 2 and the confidence factor for pruning (-C) is equal to 0.25, see Section 8.4.2 in [34] for a reference on decision trees;
- Gaussian Mixture Model (GMM): the number of Gaussians per class is taken equal to 1 in the experiments and hence each class is modeled as a multivariate Gaussian distribution (see, e.g., McLachlan and Peel [36] for a reference on Gaussian mixture modeling);
- naïve Bayes classifier (NB) from WEKA package 3.4.1 [35] with kernel estimation (-K) for modeling numeric attributes, see Section 2.12 in Duda *et al.* [34].

**Figure 5.**
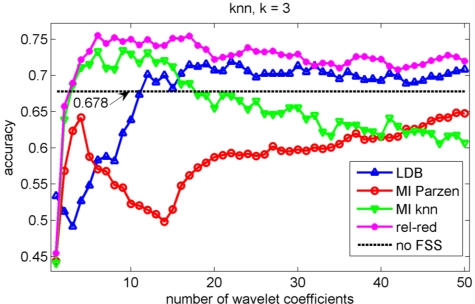
Evolution of the accuracy of the k-nearest neighbor classifier (k = 3) as a function of the number of wavelet coefficients selected with the LDB algorithm and the mutual information filter algorithms. The horizontal line indicates the accuracy when all 1,024 samples are used (no FSS).

**Figure 6.**
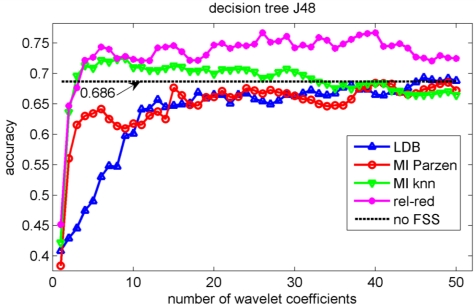
Evolution of the accuracy of the decision tree J48 classifier as a function of the number of wavelet coefficients selected with the LDB algorithm and the mutual information filter algorithms. The horizontal line indicates the accuracy when all 1,024 samples are used.

**Figure 7.**
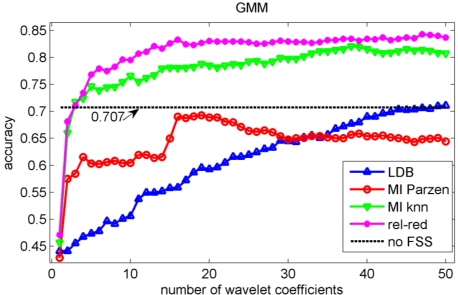
Evolution of the accuracy of the Gaussian mixture model as a function of the number of wavelet coefficients selected with the LDB algorithm and the mutual information filter algorithms. The horizontal line indicates the accuracy when the 1,024 samples were sub-sampled with a factor 15 to avoid numerical problems in the estimation of the parameters of the model. This subsampling was performed by taking the first time sample and then every 15th sample.

**Figure 8.**
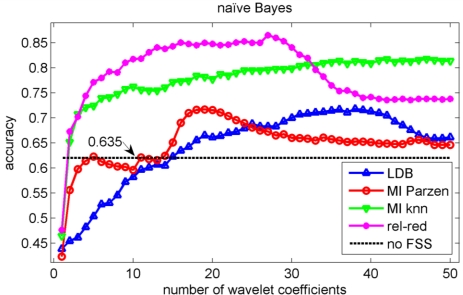
Evolution of the accuracy of naïve Bayes classifier as a function of the number of wavelet coefficients selected with the LDB algorithm and the mutual information filter algorithms. The horizontal line indicates the accuracy when all 1,024 samples are used.

In the validation of the different algorithms, we performed a 10-fold cross-validation [37]. This implies that 10 different training sets and 10 different testing sets are considered and that each data point is used once as test data in the validation. We compute the test classification performances on the sets that have not been considered in the selection of the wavelet coefficients nor in the training of the classifiers to avoid overfitting [37]. We let ‘m’, the number of selected wavelet coefficients, range from 1 to 50 coefficients. The test classification accuracies for the knn, decision tree, Gaussian mixture model and naïve Bayes classifiers are shown in Figures 5, 6, 7 and 8 respectively.

We stopped feature selection after 50 features have been selected, as can be observed from Figures 5 to 8 the testing performances of the different feature selection algorithms have leveled off at that moment. In practice, one can use a stopping rule to determine automatically how many features should be retained. This can be achieved as follows. The data is split into three parts: a training set, a validation set and a testing set. The feature selection can be stopped when the performance on the validation set does not increase further using the training set to train the machine learning algorithm. The final performance is then obtained on the testing set using the training and validation set to train the machine learning algorithm. This can be iterated in a cross-validation procedure, so that all data have been used for testing once. Note that the computational cost of feature selection algorithms will increase, because an additional validation step is included.

**Figure 9.**
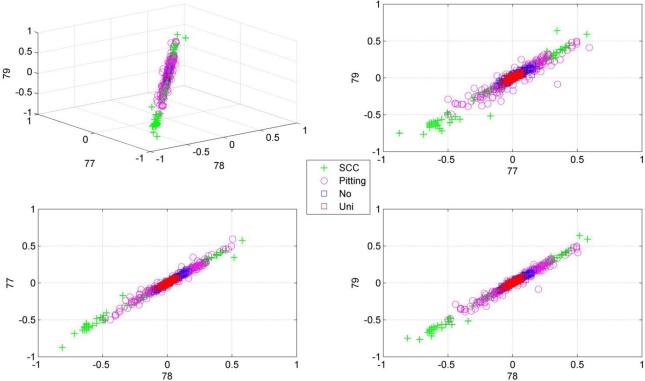
Scatter plots of the first 3 coefficients that were selected most often by the local discriminant basis algorithm (LDB) as a triplet in the 10 training sets of the 10 fold cross-validation. These are the coefficients γ_0,0,77_, γ_0,0,78_ and γ_0,0,79_ in subspace **W**_0_^0^. These scatter plots illustrate that the first three selected coefficients are highly redundant.

Note the slower increase in accuracy for the LDB algorithm compared to the mutual information approaches that can be observed in Figures 5 to 8. This is related to the fact that the LDB algorithm ignores dependencies between the wavelet coefficients. In fact, the selected wavelet coefficients are highly redundant. In each of the training folds of the 10 fold cross-validation, the local discriminant basis selection algorithm selected subspace **W**_0_^0^ as the most discriminative basis. Although the coefficients in this subspace provide discriminative information between SCC (largest values), pitting (intermediate values) and uniform corrosion + absence of corrosion (these two classes have the smallest values), the LDB algorithm was misled by the high dependencies that are present in subspace **W**_0_^0^. Indeed, in the scatter plot of Figure 9, it can be seen that the first three features, which occurred most often as a triplet in the 10 training sets of the 10 fold cross-validation, are in fact highly dependent. Each one of the three coefficients provides about the same discriminative power, so adding up their discriminative powers to obtain the joint discriminative power is misleading. The highest accuracy achieved with the LDB algorithm is obtained for the k-nearest neighbor classifier using 22 wavelet coefficients: 71.9%.

Comparison of Figures 5, 6, 7 and 8 reveals that the relevance-redundancy criterion for wavelet coefficient selection results in the highest classification accuracies. In fact, it is almost always better, no matter how many wavelet coefficients are selected, and no matter which classifier is chosen. The MI knn approach can be regarded as second best, because it is almost always better than the LDB algorithm and the MI Parzen approach in case of the decision tree, Gaussian mixture model and naïve Bayes classifiers. Note also that the performance of the relevance-redundancy approach is higher than the case when no feature subset selection (no FSS) is applied. Indeed, e.g., in Figure 8 the performance of the relevance-redundancy approach is higher than the ‘no FSS’ approach as soon as two features have been selected. In case no feature subset selection is applied, the whole signal, *i.e.*, all 1,024 time samples for each signal, are used to train the classification algorithms and to perform the predictions. The observation that a subset of features may lead to higher classification accuracies compared to the whole signal can be related to the ‘curse of dimensionality’ [34]. A part of the explanation lies in the fact that when using more features, more parameters need to be estimated for the classification algorithms based on the same finite training sample size. These parameters can only be estimated with limited accuracy, and this in turn increases the classification error. Furthermore, when using all 1,024 time samples possibly many noisy samples are included which could corrupt the prediction accuracy. One of the purposes of feature selection is to select those features from which good predictions can be generated, and ignore the noisy ones.

The classification accuracies do not reveal the structure of the errors made in the identification of the corrosion types. Therefore, we computed the confusion matrix. We concentrate on the highest accuracy we could achieve: this is obtained in Figure 8 with the naïve Bayes classifier when 27 wavelet coefficients are used. The accuracy is equal to 86.4% which is obviously much higher than could be obtained with LDB algorithm (71.9%).

The columns in the confusion matrix shown in Table 2 correspond with the known corrosion types, the rows are the predicted corrosion types using the naïve Bayes classifier. The pitting column e.g., in Table 2, shows that of all 214 pitting signals, eight are identified wrongly as absence of corrosion, 199 are identified correctly as pitting and seven are identified wrongly as SCC. This leads to a high sensitivity for pitting: 199/(8 + 7 + 199) × 100% = 93.0%. SCC can also be identified with high sensitivity: of all 205 SCC signals, six are identified wrongly as absence of corrosion, one wrongly as pitting and 198 are identified correctly. This leads to a sensitivity for SCC equal to: 198/(198 + 1 + 6) × 100% = 96.6%. Absence of corrosion and uniform corrosion are more easily (mutually) confused: the sensitivity for absence of corrosion is 73.1% and for uniform corrosion 82.0%. Note that signals from absence of corrosion and uniform corrosion are both of continuous-type emission and that their signatures in Figure 2(a–d) are hard to distinguish. It is important to note that the most harmful types of corrosion can be identified accurately, whereas the confusion between absence of corrosion and uniform corrosion is less problematic.

Finally, we note that the approach presented in this paper is generally applicable to acoustic events originating from different steel types. However, the resistance of steel towards a particular type of corrosion is influenced largely by its alloyed elements: chromium, manganese, molybdenum, nickel and nitrogen [10]. Hence, besides the acoustic activity also the steel type is indicative for the type of corrosion that is occurring. The steel type could be used as an additional discrete input variable that the machine learning algorithm can use to predict the corrosion type. Alternatively, one could use the chemical composition as an additional set of continuous input variables. However, the machine learning algorithm would require a large number of different steel types to be used in order to infer the corrosion type from the chemical composition together with the acoustic emission signals.

## 6. Conclusions

We have used the acoustic emission technique, a non-destructive testing technique, to identify different types of corrosion that occur most often in the chemical process industry. As stated in the introduction, one of the main progresses in corrosion prevention can be achieved with better information-processing strategies and the development of more efficient monitoring tools that support corrosion control programs [10]. A large progress in corrosion identification was achieved here by exploiting more advanced information-processing strategies. When the raw acoustic signals were used, the maximal accuracy achieved was rather disappointing: 70.7% (see Figure 7). A small improvement in accuracy, up to 71.9%, was achieved by using the local discriminant basis algorithm (LDB) when features are extracted with a wavelet packet decomposition. However, we noted that the LDB algorithm selected wavelet coefficients that may be highly redundant (see Figure 9). Mutual information allows us to exclude wavelet coefficients that are redundant, and this leads to a large improvement in accuracy: 86.4% using the normalized mutual information criterion and a naïve Bayes classifier. The largest confusion was observed between absence of corrosion and uniform corrosion. The most harmful corrosion types pitting and stress corrosion cracking could be indentified each with a very high sensitivity.

## Figures and Tables

**Table 1.** The steel types, the corrosive medium and the number of different experiments considered. The data was obtained from [2].

**Type of corrosion**	**Material**	**Corrosive medium + conditions**	**Number of experiments (number of time series)**	**Total number of experiments per class (number of time series)**
Absence of corrosion	1.0038	NaOH 20 weight% + NaCl 3 weight% 80 °C	1 (99)	4 (197)
	1.4541	CaCl_2_ 40 weight% 85 °C	3 (98)	
Uniform corrosion	1.0038	H_3_PO_4_ 10 weight% T_environment_	6 (194)	6 (194)
Pitting	1.4541	brackish water + FeCl_3_ 1 weight% 45 °C	9 (214)	9 (214)
Stress corrosion cracking	1.0038	Ca(NO_3_)_2_ 60 weight% 105 °C	1 (147)	10 (205)
	1.4541	CaCl_2_ 40 weight% 85 °C	9 (58)	

**Table 2.** Confusion matrix for the naïve Bayes classifier using 27 wavelet coefficients. The numbers are obtained using all 10 test folds from the 10 fold cross-validation.

	**Absence of corrosion**	**Uniform corrosion**	**Pitting**	**Stress corrosion cracking (SCC)**
**Absence of corrosion (predicted)**	**144**	35	8	6
**Uniform corrosion (predicted)**	52	**159**	0	0
**Pitting (predicted)**	1	0	**199**	1
**Stress corrosion cracking (predicted)**	0	0	7	**198**
